# Multiplex Detection of Plant Pathogens Using a Microsphere Immunoassay Technology

**DOI:** 10.1371/journal.pone.0062344

**Published:** 2013-04-26

**Authors:** Ratthaphol Charlermroj, Orawan Himananto, Channarong Seepiban, Mallika Kumpoosiri, Nuchnard Warin, Michalina Oplatowska, Oraprapai Gajanandana, Irene R. Grant, Nitsara Karoonuthaisiri, Christopher T. Elliott

**Affiliations:** 1 Institute for Global Food Security, School of Biological Sciences, Queen’s University Belfast, Belfast, Northern Ireland, United Kingdom; 2 National Center for Genetic Engineering and Biotechnology (BIOTEC), National Science and Technology Development Agency (NSTDA), Klong Luang, Pathum Thani, Thailand; Louisiana State University, United States of America

## Abstract

Plant pathogens are a serious problem for seed export, plant disease control and plant quarantine. Rapid and accurate screening tests are urgently required to protect and prevent plant diseases spreading worldwide. A novel multiplex detection method was developed based on microsphere immunoassays to simultaneously detect four important plant pathogens: a fruit blotch bacterium *Acidovorax avenae* subsp. *citrulli* (Aac), chilli vein-banding mottle virus (CVbMV, potyvirus), watermelon silver mottle virus (WSMoV, tospovirus serogroup IV) and melon yellow spot virus (MYSV, tospovirus). An antibody for each plant pathogen was linked on a fluorescence-coded magnetic microsphere set which was used to capture corresponding pathogen. The presence of pathogens was detected by R-phycoerythrin (RPE)-labeled antibodies specific to the pathogens. The assay conditions were optimized by identifying appropriate antibody pairs, blocking buffer, concentration of RPE-labeled antibodies and assay time. Once conditions were optimized, the assay was able to detect all four plant pathogens precisely and accurately with substantially higher sensitivity than enzyme-linked immunosorbent assay (ELISA) when spiked in buffer and in healthy watermelon leaf extract. The assay time of the microsphere immunoassay (1 hour) was much shorter than that of ELISA (4 hours). This system was also shown to be capable of detecting the pathogens in naturally infected plant samples and is a major advancement in plant pathogen detection.

## Introduction

Seed export is a major agricultural industry worldwide with a total of 57 countries exporting vegetable seed, accounting for 106 thousand metric tons and contributing to $2,851 million in 2010 (www.worldseed.org, last accessed in November 2012). In Thailand, specifically, export of vegetable seeds accounted for approximately 2,400 metric tons contributing to $50 million (www.worldseed.org, last accessed in November 2012). Not only are plant pathogens a serious problem for export businesses, but they may also cause disease epidemics. Conventional detection methods rely upon a symptom and morphology identification of plant disease followed by further characterization such as isolation, culturing, pathogenicity testing [1], enzyme linked immunosorbent assay (ELISA) or real-time polymerase chain reaction (PCR) [2], [3], [4]. These methods are time-consuming, laborious and require special skills such as in taxonomy to identify the pathogen responsible for disease. Therefore, an inexpensive, rapid, accurate and sensitive detection method for plant diseases is urgently required for export purpose, crop protection, plant quarantine, and disease control. To answer a current need for high-throughput screening, several multiplex detections have been developed based on molecular and immunoassay techniques. For instance, multiplex PCR assays were developed to detect multiple plant viruses such as two clades of tomato leaf curl virus (TYLCV) in tomato [5], and cucumber vein yellowing virus (CVYV) and cucurbit yellow stunting disorder virus (CYSDV) in the whitefly vector *Bemisia tabaci* [6]. These molecular techniques are sensitive but require high-skilled workers for tedious DNA extraction and purification steps which become an additional cost for sample testing. A microsphere immunoassay (xMAP technology) has emerged as an alternative for microbial detection. This technology employs different sets of fluorescence-coded microspheres (each bead set is filled with unique ratio of red/infrared dyes) conjugated with capture antibodies specific to target pathogens and the detecting antibodies are linked with another fluorophore (R-phycoerythrin, RPE). To date, there have been several reports using this technology to detect multiple analytes. For example, Luminex MagPlex microsphere was developed to detect multiple foodborne pathogens and toxin [7], three potato virus [8], and several biomarkers for potential clinical diagnostics [9], [10]. However, until now, there has never been a report on the optimization of a method to develop a multiplex plant pathogens detection using the microsphere immunoassay.

This paper is the first to describe development of an alternative immuno-based method using a microsphere immunoassay that allows multiplexing to be employed. This study describes for the first time in detail how the multiplex microsphere-based assay was optimized and used to detect the widespread plant pathogens: *Acidovorax avenae* subsp. *citruli* (Aac), chili vein-banding mottle virus (CVbMV), watermelon silver mottle virus (WSMoV) and melon yellow spot virus (MYSV). The optimized assay was validated for its accuracy and sensitivity to ensure that it will be applicable to the plant pathogens screening standard.

## Materials and Methods

### Reagents

#### Antibodies

All antibodies used in this study were obtained from the Monoclonal Antibody Production Laboratory, National Center for Genetic Engineering and Biotechnology (BIOTEC, Thailand), except for polyclonal antibody MPC which was purchased from Department of Plant Pathology, Faculty of Agriculture, Kasetsart University, Kamphaeng Saen Campus, Thailand (Table 1). The antibodies were conjugated with a fluorescent dye (R-Phycoerythrin, RPE) using a Lightning-Link™ R-Phycoerythrin conjugation kit (703–0010, Innova Biosciences, UK) or alkaline phosphatase (AP) using a Lightning-Link™ Alkaline Phosphatase Conjugation Kit (702–0010, Innova Biosciences, UK) according to manufacturers’ protocols. All labeled antibodies were kept at 4°C until use.

**Table 1:** Antibodies used in the study.

ID	Type of antibody	Specificity	Reference
**11E5**	Mouse MAb	*Acidovorax avenae* subsp. *citrulli* (Aac)	Himananto et al. 2011
**MPC**	Rabbit PAb	*Acidovorax avenae* subsp. *citrulli* (Aac)	Himananto et al. 2011
**1B4**	Mouse MAb	Potyvirus, - chilli vein-banding mottle virus (CVbMV), - watermelon mosaic virus-2 (WMV-2) - papaya ring spot virus Type W isolates (PRSV-W) - papaya ring spot virus Type P (PRSV-P) - potato virus Y (PVY)	Kumpoosiri et al. 2007
**1G8**	Mouse MAb	- chilli vein-banding mottle virus (CVbMV) - watermelon mosaic virus-2 (WMV-2) - papaya ring spot virus Type W isolates (PRSV-W) - papaya ring spot virus Type P isolates (PRSV-P) - potato virus Y (PVY)	Kumpoosiri et al. 2007
**2D6**	Mouse MAb	- capsicum chlorosis virus (CaCV) - watermelon silver mottle virus (WSMoV)	Seepiban et al. 2011
**A3**	Rabbit PAb	- capsicum chlorosis virus (CaCV) - watermelon silver mottle virus (WSMoV) - tomato necrotic ringspot virus (TNRV)	Seepiban et al. 2011
**MYSV6**	Rabbit PAb	- capsicum chlorosis virus (CaCV) - watermelon silver mottle virus (WSMoV) - tomato necrotic ringspot virus (TNRV) - melon yellow spot virus (MYSV)	Seepiban et al. 2011
**5E7**	Mouse MAb	melon yellow spot virus (MYSV)	Seepiban et al. 2011

#### Plant pathogens

A single colony of *Acidovorax avenae* subsp. *citrulli* (Aac) from a nutrient agar plate (1.5% Bacto agar, Difco #214010) was inoculated into nutrient broth (Difco, #234000) and shaken at 200 rpm for 16 h at 30^o^C. The bacterial cells were harvested by centrifugation (5000 rpm, 10 min), washed and resuspended in phosphate buffered saline (PBS) pH 7.4 containing 1 mM KH_2_PO_4_, 0.15 mM Na_2_HPO_4_, and 3 mM NaCl. The optical density (OD) was measured at 600 nm (Spectrophotometer Cintra 404) and the corresponding colony forming unit (CFU) numbers calculated using a conversion factor of 1 OD equivalent to 3×10^9^ CFU mL^−1^ by a plate count method.

For recombinant protein of virus, capsid coat protein (CP) of CVbMV and nucleocapsid protein (NP) of WSMoV and MYSV were produced to represent the plant virus during the assay optimization. PCR products of the CP and NP proteins were amplified using gene specific primers from the previously reported nucleic acid sequences (http://www.ncbi.nlm.nih.gov, GenBank accession numbers U72193, AY514625 and AY574574 for CVbMV, WSMoV and MYSV, respectively). Each purified PCR product was cloned into an expression vector, pQE80L (QIAGEN), with 6×His tag at the N-terminus of virus protein. The resulting plasmid was transformed into a designated *E. coli* host (DH5α) and the expression was induced using isopropyl-1-thio-β-D galactosidase (IPTG, final concentration of 1 mM, US biological #I8500). The 6×His-protein was purified with a Ni-NTA agarose resin column under denaturing condition. The CP and NPs of the viruses were around 30–34 kDa in weight [11], [12], [13], [14].

For leaf testing, dried leaf samples (0.08 g) were ground in 1.6 ml of 1% casein (Sigma, #C5890) in PBS containing 0.05% Tween 20 (PBST; Tween 20, Sigma #P1379), and then supernatant was collected after centrifugation at 3000 rpm at 4°C for 10 sec. Healthy plant extract was used as a negative control. The data for both ELISA and microsphere immunoassay were normalized using their corresponding negative control values.

### Microsphere Immunoassay

#### Antibodies coupling to the magnetic beads

The antibodies were coupled with specific MagPlex microsphere set (Luminex, Austin, TX) using xMAP antibody coupling kit (Luminex, #40–50016) according to the instruction manual. Briefly, the microsphere beads (1×10^6^ beads) were washed with activation buffer (Luminex, #11–25171) twice and activated by sulfo-N-hydroxysulfosuccinimide (sulfo-NHS, 50 mg mL^−1^, Luminex, #11–25169) and 1-ethyl-3-[3-dimethylaminopropyl] carbodiimide hydrochloride (EDC, 50 mg mL^−1^, Luminex, #11-40144) by shaking for 20 min at room temperature (RT). After washing excess reagent three times using a magnetic tube separator, antibody (5 µg) was added and incubated with shaking for 2 h at RT. The uncoupled antibody was removed by washing with the washing buffer (Luminex, #11-25167) using a magnetic tube separator and kept at 4°C until use.

#### Assay development

**Figure 1:**
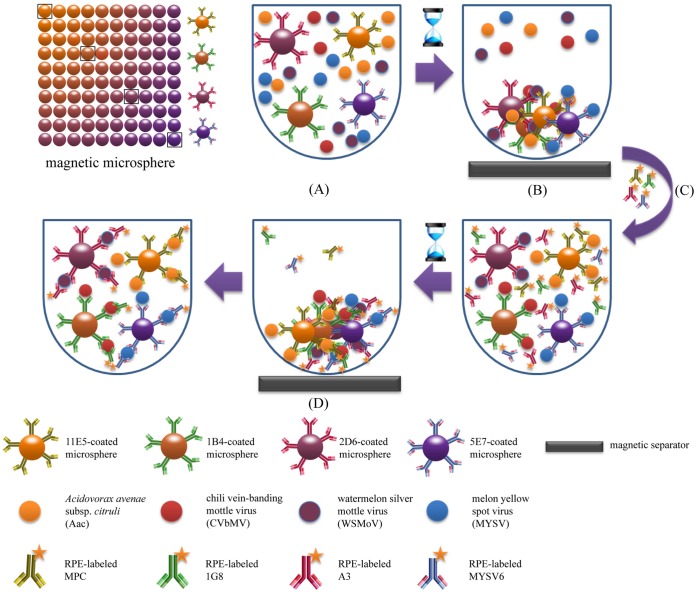
Scheme of magnetic microsphere immunoassay. (A) The specific antibody-coated microspheres were mixed samples and incubated. (B) The unbound antigens were washed and removed by using magnetic separator. (C) The cocktail of RPE-labeled antibodies was added and incubated. (D) The unbound RPE-labeled antibodies were washed and removed by using magnetic separator before signals acquired by Luminex machine.

To find the best antibody pair sets for multiplex detection, different combinations from the eight available antibodies specific to the four pathogens were tested (Table 2). The concentrations of pathogens used for the antibody pair selection experiment were 10^8^ CFU mL^−1^, 100 ng mL^−1^, 100 ng mL^−1^, and 5 µg mL^−1^ for Aac, CVbMV, WSMoV, and MYSV, respectively. Three blocking buffers, 1% skimmed milk (Difco™ laboratory, #232100), 1% casein (Sigma, #C5890) or 1% bovine serum albumin (BSA, Sigma, #A9647) in PBST were compared. A test sample (50 µl) and antibody-coated microspheres (2.5×10^3^ antibody-coated beads for each pathogen, 50 µl) were added into each well of a microplate (Greiner, #650101) and incubated for 30 min on a shaker at RT in the dark. The unbound pathogens were removed and the beads were washed with 100 µl PBST three times by using magnetic plate separator stand for 1 min each time. RPE-labeled antibodies (100 µl) at a designated concentration were added and incubated on a shaker for 30 min in the dark. After a washing step, the microspheres were resuspended with 100 µl PBST before the microspheres were detected with a red LED. The signals from RPE-labeled antibodies were measured at a 590 nm emission and a 511 nm excitation by a MAGPIX® detector (Luminex, Austin, TX) (Fig. 1). For each sample the median fluorescent intensity (MFI) value was recorded. Each test was repeated at least twice and 1% casein in PBST or healthy plant extract was used as a negative control, as appropriate.

**Table 2:** Selection of antibody pairs for the multiplex detection.

RPE-Labeled antibody		antibody coated bead							
		11E5	MPC	1G8	1B4	2D6	A3	MYSV6	5E7
	11E5	A	A						
	MPC	A	A						
	1G8			P	P				
	1B4								
	2D6					W,C	W,C	W,C	
	A3					W,C			M
	MYSV6					W,C			M
	5E7						M	M	M

*Acidovorax avenae* subsp. *citrulli* (Aac or A), potyvirus (P), watermelon silver mottle virus (WSMoV or W), capsicum chlorosis virus (CaCV or C) and melon yellow spot virus detection (MYSV or M) were used for finding the proper antibody pairs. Note: RPE is R-Phycoerythrin.

### Sandwich Enzyme-linked Immunosorbent Assay (ELISA)

For direct technical comparison with the microsphere immunoassay, the antibody sets used in sandwich ELISA were the same as those used in the microsphere immunoassay. Microtiter plates (Nunc®, #442404) were coated overnight at 4°C with 100 µl/well of a capture antibody (2.5 µg mL^−1^) diluted in 50 mM sodium carbonate-bicarbonate buffer pH 9.6. The plates were washed three times with 300 µl of PBST before blocking with 100 µl of 1% casein in PBST for 1 h at RT. The same washing steps were repeated afterwards before adding and incubating 100 µl of the pathogens at the designated concentration diluted in 1% casein in PBST for 1 h at RT. Each plate was washed before alkaline phosphatase (AP)-labeled antibody (4, 1, 2 and 2 µg mL^−1^ for MPC, 1G8, 5E7 and MYSV6, respectively) diluted in 1% casein in PBST was added and incubated for 1 h at RT. After washing step, an alkaline phosphatase substrate solution (200 µl each well) (Sigma, #P7998) was added and incubated in the dark for 1 h at RT. The absorbance at 405 nm was measured using a microplate reader (Tecan Safire2, Tecan trading AG, Switzerland).

### Gold Standard Methods for Pathogen Screening

For the Aac detection, a sandwich ELISA system is used as a gold standard method [15]. The specific protocol of this standard method is as followed. Microtiter plates were coated overnight at 4°C with 100 µl per well of a capture antibody (2.0 µg mL^−1^) diluted in sodium carbonate-bicarbonate buffer pH 9.6. The plates were washed four times with 400 µl of PBST before adding 100 µl of the pathogens diluted in extract buffer (PVPBST+: 60 mM Na_2_SO_3_, 2% wt vol^−1^ polyvinylpyrrolidone 40, 2 g L^−1^ of egg albumin and 2% Tween 20) and incubating for 1 h at RT. The same washing step was repeated before MPC antibody (4.0 µg mL^−1^) diluted in 0.5% BSA in PBST was added and incubated for 1 h at RT. Each plate was washed before goat anti-rabbit immunoglobulin (Sigma, #A3937) diluted 10,000-fold in 0.5% BSA in PBST was added for 1 h at RT. After washing step, an alkaline phosphatase substrate solution was added and incubated for 1 h. The signal was obtained from measuring absorbance at 405 nm using a Mutiskan FC Microplate Photometer reader (Thermo scientific, USA).

For CVbMV, WSMoV and MYSV screening method, a plate-trapped antigen (PTA) ELISA is commonly used a gold standard method [16]. Plant samples were ground in an extraction buffer (14 mM Na_2_SO_3_, 35 mM NaHCO_3_ and 0.2% (w/v) sodium diethyldithiocarbamate (DIECA); 5% dried weight/buffer volume or 20% wet weight/buffer volume) before 100 µL of the extracted sample was coated on each well of the microtiter plate overnight at 4°C. Each plate was washed with PBST and blocked with 2% BSA in PBST. A detecting antibody was added (100 µL each, 0.125 µg mL^−1^ of 1G8 monoclonal antibody (mAb) for CVbMV, 1.0 µg mL^−1^ of A3 polyclonal antibody (pAb) for WSMoV, and 1.0 µg mL^−1^ of 5E7 mAb for MYSV) and incubated for 1 h at RT. The washing step was repeated before alkaline phosphatase (AP) labeled goat anti-mouse immunoglobulin (Sigma, #A3562) or AP labeled anti-rabbit immunoglobulin (Sigma, #A3937) was added and incubated for 1 h at RT. After a washing step, an AP substrate solution was added and incubated for 1 h at RT. The signal was obtained from measuring absorbance at 405 nm using a Mutiskan FC Microplate Photometer reader (Thermo scientific, USA).

### Sensitivity and Assay Time

To examine how sensitivity of detection was affected by assay time, 13 concentrations of each pathogen (Aac: 1×10^2^–1×10^8^ CFU mL^−1^; CVbMV: 0.1–1000 ng mL^−1^; WSMoV: 0.5–5000 ng mL^−1^; and MYSV: 0.5–5000 ng mL^−1^) were incubated with a mixture of antibody-coated microspheres at RT and shaken for either 15, 30, 45 or 60 min before the mixture of RPE-labeled antibodies was added to detect the pathogens by incubating for either 15, 30, 45 or 60 min. The fluorescent intensities from RPE-labeled antibodies were used to fit on a dose-response curve fitting equation to obtain the limit of detection (LOD) [17], [18].
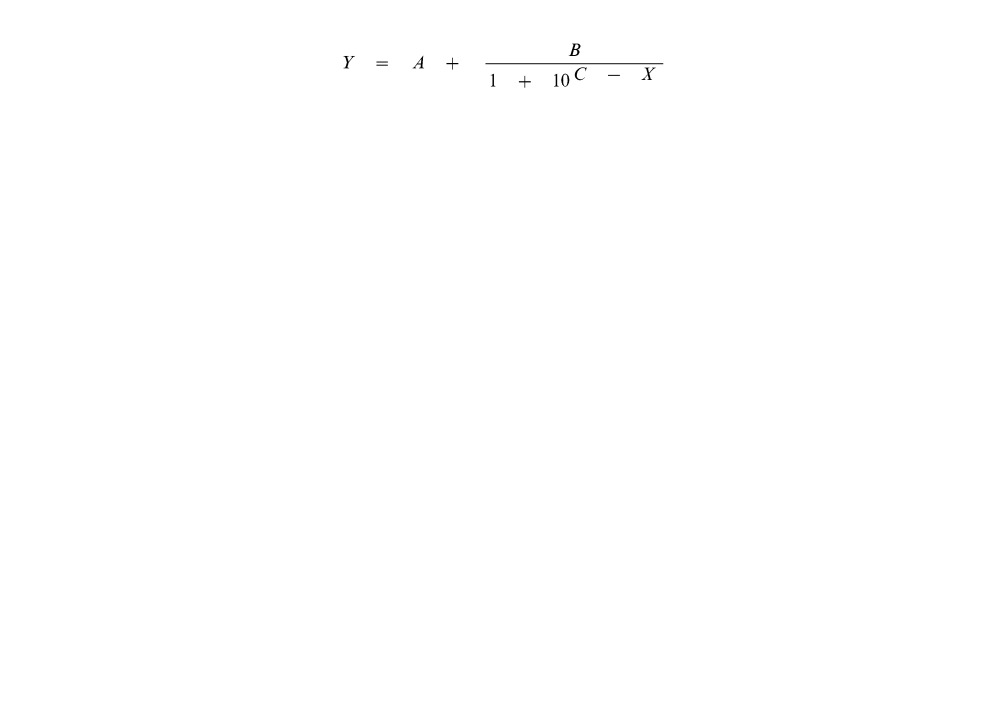



Y is the RPE fluorescent intensities when detecting pathogen concentration X, while A, B, and C are constants from curve fitting. The LOD was calculated using the intensity values greater than twice the background or negative values [19], [20]. The sensitivities of the detection by microsphere immunoassay were compared with ELISA method by using the same antibody pairs and pathogens.

### Possibility to Detect Actual Infected Plant Samples

To validate the performance of the microsphere immunoassay, naturally infected plant samples were tested and the results were compared with two methods: a sandwich ELISA and a gold standard method for each pathogen. Leaf samples known to be infected by *Acidovorax avenae* subsp. *citrulli* in watermelon (*Citrullus lanatus*), chili vein-banding mottle virus (CVbMV) in datura plant (*Datura metel*), watermelon silver mottle virus (WSMoV) and melon yellow spot virus (MYSV) were ground, diluted (no dilution, 10, 50, 100, 500, 1000-fold) in 1% casein in PBST and tested using microsphere immunoassay, sandwich ELISA and gold standard method. Leaf samples were extracted as described in plant pathogen section.

## Results and Discussion

To develop a multiplex detection of plant pathogens using a microsphere immunoassay, many factors (antibody pairs, blocking buffers, concentration of RPE-labeled antibodies) and assay time were considered during assay optimization.

### Optimization of a Microsphere Immunoassay

#### Selection of antibody pair sets for multiplex detection

To select antibody pairs for multiplex detection of *Acidovorax avenae* subsp. *citruli* (Aac), chili vein-banding mottle virus (CVbMV), watermelon silver mottle virus (WSMoV) and melon yellow spot virus (MYSV), all possible combinations of the available antibodies specific to these pathogens were coupled to different microsphere sets as capture antibodies and labeled with fluorescent R-phycoerythrin (RPE) as a detecting antibody (2.0 µg mL^−1^ of each antibody) (Table 2).

**Figure 2:**
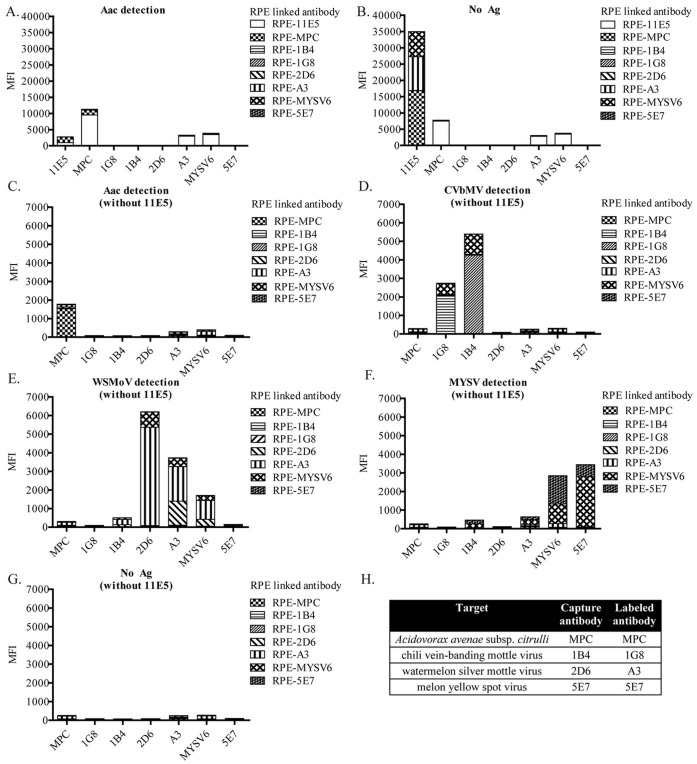
Selection of antibody pairs for the multiplex detection using a microsphere immunoassay. The detection of (A) *Acidovorax avenae* subsp. *citrulli* (Aac) and (B) no antigen using eight antibodies-coated microsphere and R-Phycoerythrin (RPE) labeled antibodies, including 11E5 antibody. The detection of (C) Aac, (D) chilli vein-banding mottle virus (CVbMV), (E) watermelon silver mottle virus (WSMoV), (F) melon yellow spot virus (MYSV) detection and (G) no antigen with seven antibodies- coated microsphere and RPE-labeled antibodies without using 11E5 antibody. X-axis is antibody-coated microsphere and y-axis is median fluorescent intensity (MFI) from each RPE-labeled antibody. (H) Summary of selected antibody pair sets for the detection of the four plant pathogens.

Although specificity and cross-reactivity of these antibodies were previously characterized by ELISA [15], [16], [21], their specificity in a multiplex detection using a microsphere immunoassay has never been tested. A previous study suggested that not all antibodies that are compatible to an ELISA format will be readily transferable to the microsphere immunoassay [22]. Therefore, it was vital to select appropriate pairs of these antibodies to be used in the system. For Aac detection, RPE-labeled 11E5 cross reacted with A3- and MYSV6-coated microspheres (Fig. 2A). Considering the negative control (no antigen), 11E5-coated microsphere and RPE-labeled 11E5 caused non-specific binding with MPC, A3 and MYSV6 in this microsphere immunoassay (Fig. 2B) whereas this cross-reactivity between 11E5 and MPC was not observed previously in a sandwich ELISA format [15]. On the other hand, MPC-coated microsphere and RPE-labeled MPC were highly specific to Aac without non-specific binding (Fig. 2C). For CVbMV detection, 1G8 and 1B4 were tested and it was found that 1G8- and 1B4-coated microspheres could pair with RPE-labeled 1B4 and 1G8, respectively, in this assay format. However, signal from a pair of 1B4-coated microsphere and RPE-labeled 1G8 was higher than that from a pair of 1G8-coated microsphere and RPE-labeled 1B4; therefore, 1B4-coated microsphere and RPE-labeled 1G8 were chosen for the CVbMV detection. In addition, RPE-labeled MYSV6 was found to cause cross-reactivity with 1G8- and 1B4-coated microspheres in the CVbMV detection system, thus, MYSV6 could not be used for the multiplex detection (Fig. 2D). For WSMoV detection, 2D6-coated microsphere and RPE-labeled A3 gave higher signal than other antibody sets, thus, they were chosen for this detection. For MYSV detection, the pairing of 5E7-coated microsphere and RPE-labeled 5E7 was the only option (Fig. 2F) because RPE-labeled MYSV6 caused cross-reactivity in the CVbMV system (Fig. 2D) and MYSV6-coat microsphere caused non-specific binding with RPE-labeled A3 (Fig. 2G). Moreover, using these selected antibody sets resulted in low background (Fig. 2G). Therefore, the selected antibody sets were used in the subsequent experiments (Fig. 2H). The antibody pair selection result indicates that although some antibodies can be used for a single detection, they might cause cross reactivity with other antibodies used in a multiplex detection. The cross-reactivity in multiplex format might be explained by the specificity of the antibodies to the targets whose affinity of antibodies depends on the heterogenicity and hydrophobicity of amino acid of antibodies [23], [24]. Since a sandwich ELISA requires pairing between capture and secondary antibodies, the selection of appropriate antibody pairs is important and a crucial requirement for multiplex detection [25].

#### Blocking buffer selection and optimization of RPE-labeled antibody concentrations

**Figure 3:**
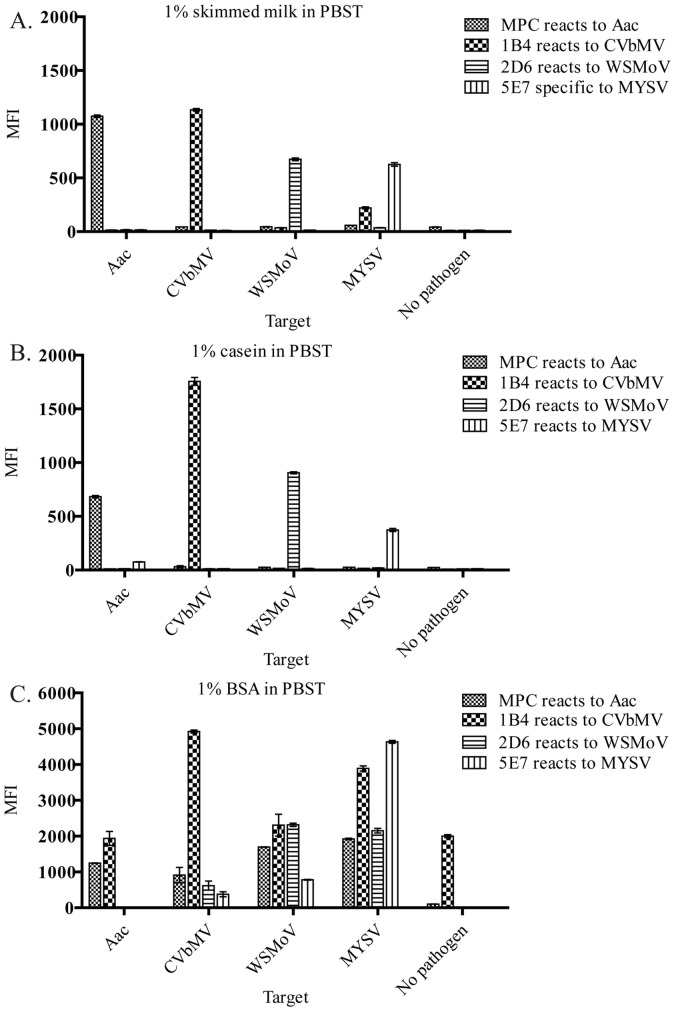
Selection of blocking buffers. A mixture of antibody-coated microsphere, MPC react to *Acidovorax avenae* subsp. *citrulli* (Aac), 1B4 specific to chilli vein-banding mottle virus (CVbMV), 2D6 specific to watermelon silver mottle virus (WSMoV) and 5E7 specific to melon yellow spot virus (MYSV), was tested with a single antigen and no pathogen using (A) 1% skimmed milk, (B) 1% casein or (C) 1% bovine serum albumin (BSA) as the blocking agent. Mixture of RPE-labeled antibodies, MPC, 1G8, A3 and 5E7, were used as a detecting system for Aac, CVbMV, WSMoV and MYSV, respectively. Y-axis is median fluorescent intensity (MFI). Each dataset was plotted as a mean of duplicates ± standard deviation.

Three blocking buffers, 1% skimmed milk, 1% casein and 1% BSA in PBST, were compared for their ability to prevent non-specific binding. 1% skimmed milk gave highly specific detection for all pathogens except for MYSV where non-specific signals were obtained on capture antibodies for CVbMV and WSMoV (1B4- and 2D6-coated microsphere, respectively; Fig. 3A). On the other hand, 1% casein as a blocking buffer was able to reduce non-specific binding in all detections. Fluorescent intensities of CVbMV and WSMoV detections increased about 1.3–1.5 times whereas those of Aac and MYSV reduced about 1.5–1.7 times when compared to that of a negative control using 1% skimmed milk as a blocking buffer (Fig. 3B). Although casein is a milk protein, there are many additional proteins in milk that might bind non-specifically to RPE-labeled 1B4 when skimmed milk was used as a blocker. For 1% BSA as a blocking buffer, not only was a high signal from the background (no pathogen) observed, but it also gave non-specific binding signals in all detections (Fig. 3C). Although BSA is commonly used to prevent non-specific binding from hydrophobic interaction between protein and ionic or electrostatic interactions [26], it did not seem to help prevent non-specific binding or lower the background signal in our study. Therefore, 1% casein was selected as a blocking buffer to optimize concentrations of RPE-labeled antibodies in subsequent experiments. From our previous experiences with other immunoassay formats, it is very important to perform experiments to select the most effective blocking buffer for each assay. For instance, skimmed milk was the best blocking reagent whereas BSA resulted in a high background for a foodborne pathogen antibody array [27]. On the other hand, both skimmed milk and BSA were found to be the most effective blockers for an antibody for hybridoma screening [28]. In addition, several commercially available blocking buffers were evaluated and shown to be effective in eliminating non-specific binding in a microsphere immunoassay [29].

To obtain high signal and sensitivity, concentrations of RPE-labeled antibodies ranging between 0.5–8.0 µg mL^−1^ were examined. The optimal concentrations of RPE-labeled MPC, 1G8, A3 and 5E7 were 8.0, 2.0, 0.5 and 4.0 µg mL^−1^, respectively (data not shown). Optimal concentration of RPE-labeled antibody is crucial for this assay development. Using too high concentration of the antibody would result in non-specific binding while too low concentration would result in low signal and low sensitivity.

### Multiplex Detection

**Figure 4:**
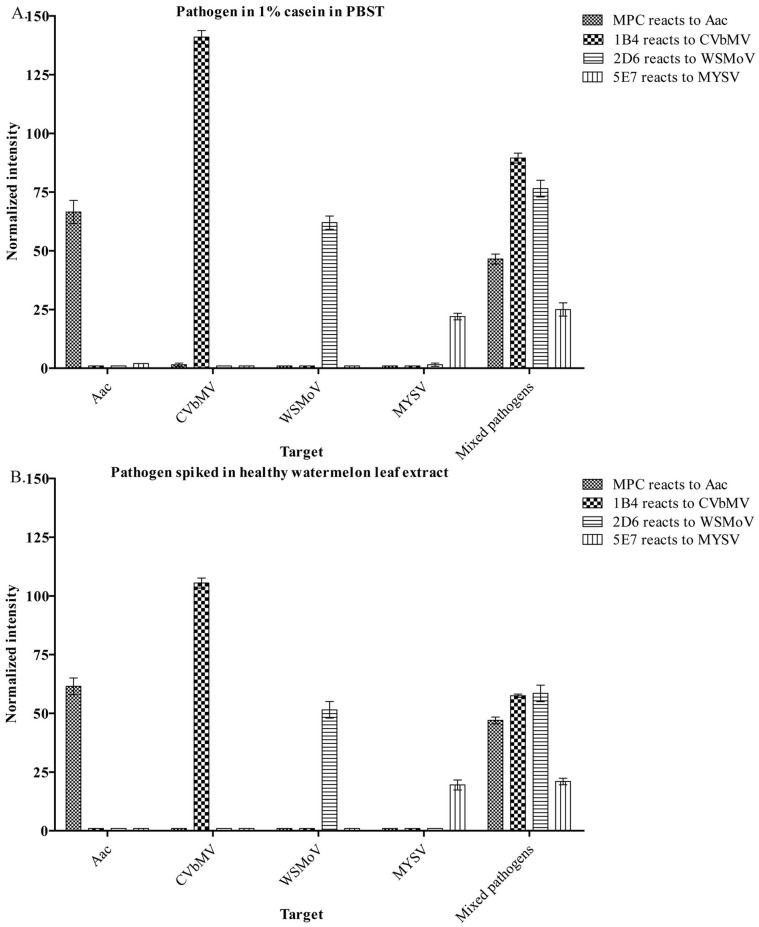
Multiplex detection of four plant pathogens. *Acidovorax avenae* subsp. *citrulli* (Aac) (10^8^ CFU mL**^−^** ^1^), chilli vein-banding mottle virus (CVbMV) (0.2 µg mL**^−^** ^1^), watermelon silver mottle virus (WSMoV) (5 µg mL**^−^** ^1^), melon yellow spot virus (MYSV) (10 µg mL**^−^** ^1^) and mixed pathogens (10^8^ CFU mL**^−^** ^1^) Aac, 0.2 µg mL**^−^** ^1^ CVbMV, 5 µg mL**^−^** ^1^ WSMoV and 10 µg mL**^−^** ^1^ MYSV) in (A) 1% casein in PBST and (B) artificially spiked healthy watermelon leaf extract were tested using immuno microsphere. Antibody (MPC, 1B4, 2D6 and 5E7) coated microspheres were used to detect Aac, CVbMV, WSMoV and MYSV, respectively. Normalized signal (Y-axis) is a ratio of signal obtained from pathogen detection in the samples to the signal obtained when no pathogen was present. Each dataset was plotted as a mean of duplicates ± standard deviation.

Once the optimal conditions had been obtained, the capability for multiplex detection was examined by simultaneously detecting four plant pathogens (1×10^8^ CFU mL**^−^** ^1^ Aac, 0.1 µg mL**^−^** ^1^ CVbMV, 5 µg mL**^−^** ^1^ WSMoV and 5 µg mL**^−^** ^1^ MYSV) when diluted in a blocking buffer (1% casein in PBST) or spiked into healthy watermelon extract. In the buffer, single and mixed pathogens detections gave accurate results though signals of Aac and CVbMV in multiplex detection were lower than those of single detections (Fig. 4A). For the detection of pathogens spiked in healthy watermelon leaf extract, the results were similar to those in the buffer. For example, the signal of CVbMV detection spiked in plant extract in the single detection was lower than that in the buffer about 1.8 times; however, our system still gave accurate detection in both single and multiplex formats (Fig. 4B). This result demonstrates that the system can detect pathogens spiked in healthy watermelon leaf extract. The lower signal of Aac and CVbMV detection in a multiplex detection than that in a single detection could be due to interference from other non-target bacteria or viruses in test samples [25].

### Sensitivity of Detection and Assay Time

**Figure 5:**
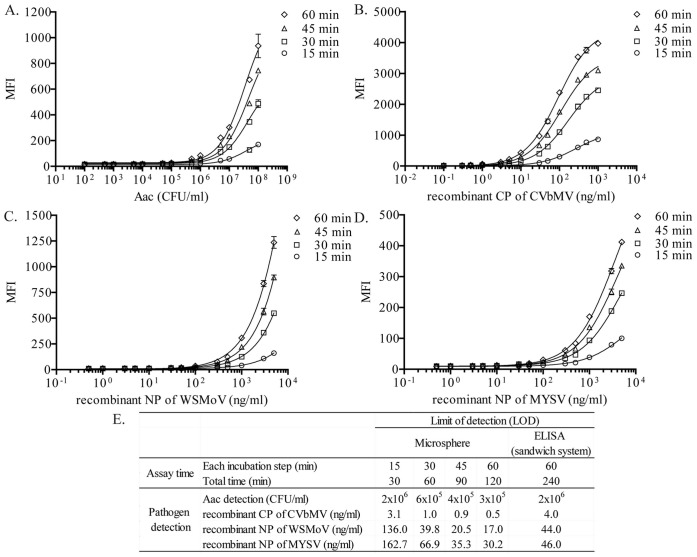
Effects of assay time on sensitivity of detection. The different concentrations of *Acidovorax avenae* subsp. *citrulli* (Aac) (A), recombinant coat protein (CP) of chilli vein-banding mottle virus (CVbMV) (B), recombinant nucleocapsid protein (NP) of watermelon silver mottle virus (WSMoV) (C) and melon yellow spot virus (MYSV) (D) were detected in the microsphere immunoassay using four incubation times: 15 min (circle), 30 min (square), 45 min (triangle) and 60 min (diamond). Y-axis is a median fluorescent intensity (MFI). Each data point was plotted as a mean of duplicates ± standard deviation. (E) Comparison of sensitivity of detection between microsphere immunoassay (four different incubation times) and sandwich ELISA (60 min incubation only) with the same sets of antibodies.

Sensitivity of detection using a microsphere immunoassay was compared with that of sandwich ELISA method using the same sets of antibodies. Moreover, in order to find the shortest assay time without compromising sensitivity of detection using the microsphere immunoassay, four incubation times (15, 30, 45 or 60 min) were examined. Increasing incubation times between tested samples and antibody-coated microspheres slightly improved sensitivities in all cases of detection (Fig. 5A–D). To obtain the same LOD as the sandwich ELISA, at least 30 minutes are required for an incubation step of the microsphere immunoassay. However, if 60 minutes were used for an incubation step, the detection by the microsphere immunoassay gave 10, 8, 2.6 and 1.5 times better sensitivity for Aac, CVbMV, WSMoV and MYSV detection, respectively, than by the sandwich ELISA. The microsphere immunoassay method is more sensitive than the sandwich ELISA method when at least 45 minutes were used for each incubation step. Therefore, only one hour of a total assay time for the microsphere immunoassay was required to achieve the same sensitivity as ELISA method which required four hours of a total assay time (Fig. 5E). In a previous report, paramagnetic microspheres were used to detect potato virus X (PVX), potato virus Y (PRY) and potato leafroll virus (PLRV), and the sensitivity of PVX and PLRV detection was about 10 times higher than ELISA; however, the sensitivity of PRY detection was less than ELISA [8]. In our study, the optimized conditions of the microsphere immunoassay seemed to help improving detection sensitivity for all pathogens from those obtained from the sandwich ELISA. The better sensitivity might be explained from the fact that the microsphere immunoassay is a fluorescent-based detection while the ELISA is chromogenic detection. Previously, fluorescent-based detections were reported to be more sensitive than chromogenic detection. For instance, the sensitivity of alkaline phosphatase increased by 6–13 times when using fluorogenic substrate (4-methylumbelliferyl phosphate; 4MeUP) instead of phenolphthalein monophosphate (PMP) and *p*-nitrophenyl phosphate (pNPP) which are chromogenic substrates in time-resolved fluoroimmunoassay [30].

### Plant Pathogen Detection in Naturally Infected Samples

To validate the accuracy of the microsphere immunoassay, naturally infected leaf samples (Aac-infected watermelon leaves, CVbMV-infected in datura leaves, WSMoV and MYSV-infected in watermelon leaves) were tested. The samples were diluted at different dilution factors (no dilution, 10, 50, 100, 500 and 1000 times) and tested by three methods: the microsphere immunoassay, sandwich ELISA with the same antibody set as those in the microsphere immunoassay, and the gold standard method for each pathogen. For each method, the signal from tested sample was normalized by the signal from the negative controls which were corresponding healthy leaf extracts. The results from the three systems were in agreement but with different sensitivities (Table S1). For Aac detection, the microsphere immunoassay was able to detect at the lowest detection limit (at 100-fold dilution) whereas the gold standard method (sandwich ELISA with 11E5 and MPC antibody pair) and sandwich ELISA method (MPC and MPC antibody pair) could detect Aac infected plant up to 50-fold dilution (Table S1A). For CVbMV detection, the gold standard method was a plate-trapped antigen ELISA which was still able to detect CVbMV-infected plant at a 1000-fold dilution, whereas the sandwich ELISA and the microsphere immunoassay could detect at 10 and 50 diluted times, respectively (Table S1B). For WSMoV and MYSV detection, results were similar to CVbMV detection in that the gold standard method (PTA-ELISA) gave a higher sensitivity than the sandwich ELISA and the microsphere immunoassay method (Table S1C-D). This result is not surprising because a sandwich ELISA system often gave lower sensitivity than plate-trapped antigen ELISA [31]. However, it has been reported that the plate-trapped antigen ELISA was highly sensitive to interference from crude plant sap extract [32], thus, a sandwich ELISA system often becomes an alternative with its short assay time without having to coat the sample on the plate. When comparing between the sandwich ELISA and the microsphere immunoassay with the same sets of antibodies used in this study, the microsphere immunoassay always gave higher sensitivity in the detection of pathogens infected in plant samples. Additionally, three dimensional suspension-based immunoassay such as that used in this study helps reducing interference from the sample matrix by providing better separation of proteins from plant extract and removal of non-interest targets during magnetic separation [33]. The feasibility to employ the microsphere immunoassay directly without complicated sample preparation was proven in this study. The results that the microsphere immunoassay was able to detect pathogens in naturally infected samples with a higher signal than the sandwich ELISA method make it a very promising alternative method for plant pathogen screening technique.

### Conclusion

The optimization of numerous factors in relation to a multiplex microsphere immunoassay was successful for plant pathogens detection. One big advantage of using magnetic microsphere immunoassay is the fact that it helps capture the pathogens out of the interfering components in the sample matrix. The microsphere immunoassay developed in this study achieved better sensitivity of detection than a sandwich ELISA method if the same antibody sets were used and its assay time is also shorter. With the optimal assay conditions, the microsphere immunoassay was demonstrated to be able to detect multiple pathogens accurately even in naturally infected plant samples. The capacity of this microsphere immunoassay technique could be further expanded to higher throughput such as detecting up to 50 targets simultaneously. The system could also become fully automatic if dealing with a larger volume of routine testing. In addition, the details of assay development from this study will help others optimizing similar multiplex detection using magnetic microsphere immunoassays for different purposes in the future.

## Supporting Information

Table S1 **Plant pathogen detection in real infected samples.** (A) *Acicidovorax avenae* subsp. *citrulli* (Aac) infected in watermelon (*Citrullus lanatus*), (B) chili vein-banding mottle virus (CVbMV) infected *in Datura metel*, (C) watermelon silver mottle virus (WSMoV) and (D) melon yellow spot virus (MYSV) infected in watermelon were diluted by six different dilution factors (DF) (1, 10, 50, 100, 500 and 1000-fold) and tested using three different assay formats (gold standard method, sandwich ELISA and microsphere immunoassay (MIA)). The gold standard method for the Aac detection was a sandwich ELISA where 11E5 is a capture antibody and MPC is a secondary antibody. The gold standard method for the CVbMV, WSMoV and MYSV detection is a plate-trapped antigen (PTA) ELISA with designated antibodies. MIA is a microsphere immunoassay. The signals obtained from the pathogen detection were normalized to the signal obtained from the detection in watermelon or *Datura metel*. The value from normalization was considered as a positive result (+) when it was above at least twice of background interpreting.
(DOCX)
