# Supplementary material for: Multiplex Detection of Plant Pathogens Using a Microsphere Immunoassay Technology
Source: PLoS One. 2013 Apr 26;8(4):e62344. doi: 10.1371/journal.pone.0062344 (PMC3637204; doi:10.1371/journal.pone.0062344)
Supplement: Supplementary file 1 [file pone.0062344.s001.docx]

A.

DF	Sample: Aac infected watermelon											
	Aac detection			CVbMV detection			WSMoV detection			MYSV detection		
	Gold Std.	Sandwich	MIA	Gold Std.	Sandwich	MIA	Gold Std.	Sandwich	MIA	Gold Std.	Sandwich	MIA
	11E5-MPC	MPC-MPC	MPC-MPC	PTA, 1G8	1B4-1G8	1B4-1G8	PTA, A3	2D6-A3	2D6-A3	PTA-5E7	5E7-5E7	5E7-5E7
1	+	+	+	-	-	-	-	-	-	-	-	-
10	+	+	+	-	-	-	-	-	-	-	-	-
50	+	+	+	-	-	-	-	-	-	-	-	-
100	-	-	+	-	-	-	-	-	-	-	-	-
500	-	-	-	-	-	-	-	-	-	-	-	-
1000	-	-	-	-	-	-	-	-	-	-	-	-

B.

DF	Sample: CVbMV infected in *Datura metel*											
	Aac detection			CVbMV detection			WSMoV detection			MYSV detection		
	Gold Std.	Sandwich	MIA	Gold Std.	Sandwich	MIA	Gold Std.	Sandwich	MIA	Gold Std.	Sandwich	MIA
	11E5-MPC	MPC-MPC	MPC-MPC	PTA, 1G8	1B4-1G8	1B4-1G8	PTA, A3	2D6-A3	2D6-A3	PTA-5E7	5E7-5E7	5E7-5E7
1	-	-	-	+	+	+	-	-	-	-	-	-
10	-	-	-	+	+	+	-	-	-	-	-	-
50	-	-	-	+	-	+	-	-	-	-	-	-
100	-	-	-	+	-	-	-	-	-	-	-	-
500	-	-	-	+	-	-	-	-	-	-	-	-
1000	-	-	-	+	-	-	-	-	-	-	-	-

C.

DF	Sample: WSMoV infected in watermelon											
	Aac detection			CVbMV detection			WSMoV detection			MYSV detection		
	Gold Std.	Sandwich	MIA	Gold Std.	Sandwich	MIA	Gold Std.	Sandwich	MIA	Gold Std.	Sandwich	MIA
	11E5-MPC	MPC-MPC	MPC-MPC	PTA, 1G8	1B4-1G8	1B4-1G8	PTA, A3	2D6-A3	2D6-A3	PTA-5E7	5E7-5E7	5E7-5E7
1	-	-	-	-	-	-	+	+	+	-	-	-
10	-	-	-	-	-	-	+	+	+	-	-	-
50	-	-	-	-	-	-	+	+	+	-	-	-
100	-	-	-	-	-	-	+	+	+	-	-	-
500	-	-	-	-	-	-	+	-	+	-	-	-
1000	-	-	-	-	-	-	+	-	-	-	-	-

D.

DF	Sample: MYSV infected in watermelon											
	Aac detection			CVbMV detection			WSMoV detection			MYSV detection		
	Gold Std.	Sandwich	MIA	Gold Std.	Sandwich	MIA	Gold Std.	Sandwich	MIA	Gold Std.	Sandwich	MIA
	11E5-MPC	MPC-MPC	MPC-MPC	PTA, 1G8	1B4-1G8	1B4-1G8	PTA, A3	2D6-A3	2D6-A3	PTA-5E7	5E7-5E7	5E7-5E7
1	-	-	-	-	-	-	-	-	-	+	+	+
10	-	-	-	-	-	-	-	-	-	+	-	+
50	-	-	-	-	-	-	-	-	-	+	-	-
100	-	-	-	-	-	-	-	-	-	+	-	-
500	-	-	-	-	-	-	-	-	-	+	-	-
1000	-	-	-	-	-	-	-	-	-	+	-	-
